# Validating DigiHEALTHQUAL for measuring digital health effects on service quality in outpatient care in Germany

**DOI:** 10.1038/s41746-026-03302-0

**Published:** 2026-10-02

**Authors:** Dominik Rigo, Leonard Fehring, Achim Mortsiefer, Sven Meister

**Affiliations:** 1 Health Care Informatics, Faculty of Health, Department of Medicine, Witten/Herdecke University, Witten, Germany; 2 General Practice II and Patient-Centeredness in Primary Care, Faculty of Health, Department of Medicine, Witten/Herdecke University, Witten, Germany; 3 Department of Health Care, Fraunhofer Institute for Software and Systems Engineering ISST, Dortmund, Germany

**Keywords:** Health care, Medical research, Psychology, Psychology

## Abstract

The adoption of digital health solutions (DHS) in outpatient care is hindered by uncertainty regarding their non-clinical effects and a lack of validated instruments for standardized evaluation. Health care service quality (HCSQ) provides a theoretically grounded framework for assessing these effects, but no validated HCSQ-based instrument has been established. This study aimed to quantitatively validate DigiHEALTHQUAL and compare a theory-derived model structure with a data-derived alternative. We conducted a cross-sectional online survey among 1085 adult patients in Germany with experience using one of four DHS: online appointment booking, digital anamnesis, video consultation, or electronic health records. Psychometric validation included internal consistency analyses, confirmatory factor analysis (CFA), exploratory factor analysis (EFA), and comparative model evaluation. The refined theory-derived seven-factor model excluding general satisfaction demonstrated good model fit and strong internal consistency, although discriminant validity between dimensions was limited. EFA yielded a more parsimonious four-factor structure with a dominant process-related factor. High inter-factor correlations supported a higher-order HCSQ structure. Overall, DigiHEALTHQUAL showed validity for assessing non-clinical DHS effects on HCSQ in outpatient care in Germany and supports standardized DHS evaluation in research and practice.

## Introduction

We understand digital health solutions (DHS) as “information and communications technologies in medicine […] to manage illnesses and health risks and to promote wellness”^1^. DHS are widely considered to hold substantial potential for transforming health care delivery. Prior research has shown that DHS can improve clinical outcomes^2^, but also non-clinical aspects of health care delivery, such as the overall patient experience and service quality. For instance, DHS have been shown to enhance accessibility^3^, improve organizational processes^4^, and reduce costs^5,6^. Despite these advantages and a rapidly growing availability of DHS^7^, adoption in outpatient care remains comparatively slow. This is partly due to the structure of outpatient care with its typically smaller practices and limited organizational resources, for instance no dedicated IT or procurement units^8,9^.

A central barrier to DHS adoption is the comparison of the efforts and costs for the implementation and maintenance against the effects of DHS^10,11^, which is a central consideration for practice owners and managers to select DHS for their practices as well as for payers and policymakers assessing DHS strategies. A substantial body of research has examined the effects of DHS; however, two patterns can be observed. First, many studies - mostly studies related to non-clinical effects - rely on self-developed, non-validated instruments with unclear psychometric properties^4,5,12–14^. Second, validated instruments are commonly applied in studies related to the clinical outcomes, but tend to be narrowly focused on specific DHS, therapeutic areas, or care settings^15–17^. Without a broad consensus instrument, existing evidence remains fragmented and comparability across studies limited, particularly for the systematic assessment of DHS effects beyond clinical outcomes.

Many of the non-clinical effects of DHS relate to how care is organized, delivered, and experienced by patients and health care professionals. Such aspects are commonly conceptualized in academia as dimensions of health care service quality (HCSQ). Donabedian’s HCSQ framework distinguishes between structure, process, and outcome, thereby going beyond the clinical aspects of health care delivery^18^. In line with Donabedian, we understand HCSQ as patients’ and health care professionals’ holistic perceptions of health care delivery based on the comparison between expectations and perceived performance^19^.

HCSQ is a well-established concept with a strong methodological foundation and has been evaluated using validated instruments, many of which are based on service quality models such as SERVQUAL^20,21^. Importantly, core dimensions of different HCSQ models overlap with domains that DHS are specifically designed to influence^19^, for instance, therapy adherence^22^, efficiency^4^, or patient-provider interaction^23^. This conceptual overlap suggests that HCSQ offers a theoretically grounded lens for evaluating DHS effects beyond clinical outcomes. Yet, despite this overlap, no validated HCSQ-based instrument has been systematically applied to assess DHS-related effects in outpatient care.

To address this gap, we used DigiHEALTHQUAL, a questionnaire based on three validated HCSQ instruments. DigiHEALTHQUAL was designed as a generic, use-case-spanning instrument for post-implementation assessment of the perceived effects of DHS on HCSQ. It is intended to compare different DHS in outpatient care at the level of service-quality dimensions rather than to evaluate a specific product, vendor, or technology^24^. The present study evaluates DigiHEALTHQUAL across four highly relevant, patient-facing DHS (online appointment booking, digital anamneses, video consultation, and electronic health records) in outpatient care settings in Germany, including general and specialist practices. While DigiHEALTHQUAL builds on established instruments, it represents a synthesis of multiple models and, in its current form, has not yet been empirically validated as a unified factor structure. Therefore, the primary aim was to conduct a quantitative psychometric validation of DigiHEALTHQUAL with patients in real-world outpatient settings. The secondary aim was to compare the theory-derived DigiHEALTHQUAL model with the data-derived DigiHEALTHQUAL model.

## Results

### Sample characteristics

A total of 3725 responses from patients were collected. After applying predefined data cleaning procedures, 1454 responses were identified as eligible, of which 1085 remained for analysis after additional quality assurance and privacy control filters (Supplementary Fig. 1). Participating patients were predominately male (662/1085, 61%), aged 55–65 years (264/1085, 24%), and most commonly lived in places of residence with 20,001–100,000 inhabitants (300/1085, 28%). The majority was insured under statutory health insurance (841/1085, 78%) and reported feeling rather digitally savvy (488/1085, 45%; Suppl. Fig. 2). The completion time for the final survey was approximately 6 minutes (median) and 9 minutes (mean).

### Exploratory factor analysis for a data-derived model

Applying the predefined item reduction rules (removal of non-loading, cross-loading, and low-loading items), 20 iterative exploratory factor analysis (EFA) steps were required to derive a four-factor model comprising 32 items. 19 out of 51 items were removed: EF8, GS2, TA2, and TA4 were non-loading items; EF7, GS1, SA1-7, TA1, TA3, and TA5 were cross-loading items; and EF6, EM6, and EM9 were low-loading items. No further item removal was justified after the final iteration.

Bartlett’s test of sphericity and the Kaiser-Meyer-Olkin measure indicated excellent suitability for EFA (Bartlett’s test *p* < 0.001; Kaiser-Meyer-Olkin measure range 0.98–0.99). Four factors were retained based on eigenvalues ≥ 1 and scree plot inspection (Supplementary Table 1): information and efficiency (11 items), accessibility (7 items), empathy (7 items), and degree of improvement of care services (7 items), explaining 58%, 4%, 3%, and 2% of the variance, respectively. Inter-factor correlations ranged from 0.64 to 0.83 (Suppl. Table 2). Information and efficiency formed a dominant, process-related factor, while accessibility, empathy, and degree of improvement of care services remained empirically distinct and supported by existing HCSQ frameworks^24^. This pattern suggests a potential higher-order organization of HCSQ dimensions. General satisfaction, tangibles, and safety did not emerge as separate factors, potentially linked to ceiling effects and restricted variance of several of their items.

### Confirmatory factor analysis of the data-derived model

**Fig. 1:**
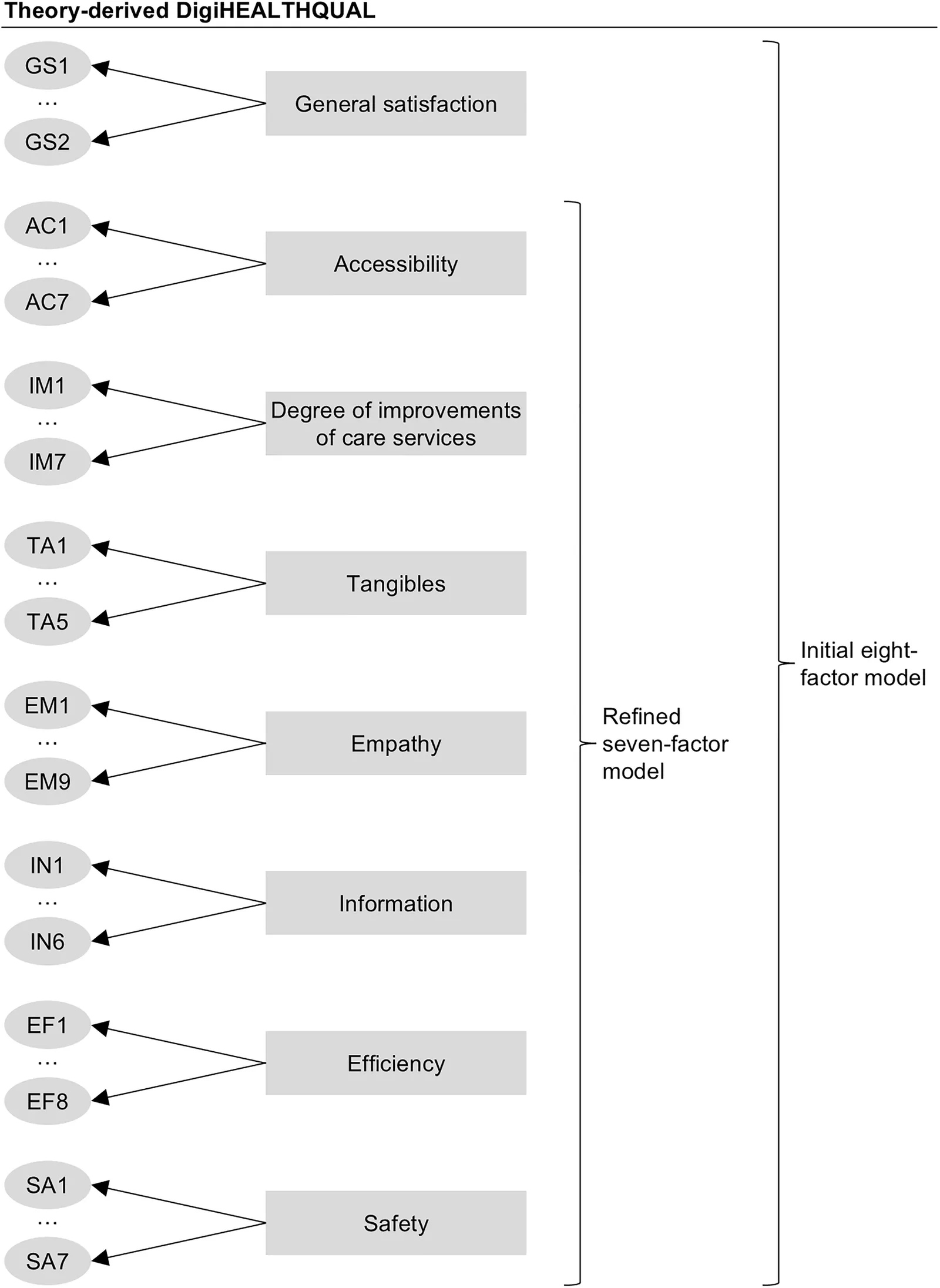
Data-derived DigiHEALTHQUAL models evaluated using confirmatory factor analysis. The data-derived DigiHEALTHQUAL instrument comprises 32 items across four dimensions: information and efficiency (11 items), accessibility (7 items), empathy (7 items), and degree of improvement of care services (7 items). Confirmatory factor analysis assessed a first-order model and a higher-order model with healthcare service quality as overarching second-order factor as well as an unidimensional model with all items loading on healthcare service quality.

The obtained four-factor model was evaluated using confirmatory factor analysis (CFA) with a final sample of *N* = 843 (Fig. 1). All items demonstrated acceptable standardized factor loadings ranging from 0.71 to 0.88. Squared multiple correlations ranged from 0.53 to 0.78, indicating that a substantial proportion of item variance was explained by the respective latent factors. These results indicate good indicator reliability and strong measurement quality across all four dimensions (Suppl. Table 3).

Inter-factor correlations in the first-order model were high, ranging from 0.73 to 0.88 (Table 1), reflecting strong associations between HCSQ dimensions. Internal consistency estimates were high for all factors. Cronbach’s α values ranged from 0.90 to 0.96, indicating excellent internal consistency, but also potential item redundancy, which is supported by the high values for average inter-item correlation and corrected item-total correlations. For all four factors, composite reliability (CR) values were acceptable and identical to Cronbach’s α. This convergence reflects high and similar factor loadings within each dimension, a condition under which Cronbach’s α and CR yield comparable estimates.

**Table 1:** Confirmatory factor analysis: Quality assessment of the data-derived DigiHEALTHQUAL model

Construct	Accessibility	Degree of improvement of care services	Empathy	Information & efficiency
Accessibility	—			
Degree of improvement of care services	0.76	—		
Empathy	0.77	0.84	—	
Information & efficiency	0.73	0.88	0.88	—
Cronbach’s α	0.90	0.94	0.95	0.96
CR	0.90	0.94	0.95	0.96
Average inter-item correlation	0.56	0.68	0.73	0.69
Corrected item-total correlation (range)	0.66–0.73	0.78–0.82	0.79–0.86	0.76–0.86
AVE	0.56	0.73	0.68	0.69
√AVE	0.75	0.86	0.83	0.83
HTMT (range)	0.74–0.77	0.77–0.88	0.76–0.88	0.74–0.88

Inter-factor correlation matrix of the obtained four DigiHEALTHQUAL factors; reliability assessment using Cronbach’s α, composite reliability (CR), average inter-item correlation and corrected item-total correlation, and convergent and discriminant validity assessment based on average variance extracted (AVE), √AVE, and heterotrait-monotrait ratios (HTMT).

All constructs demonstrated adequate convergent validity, with average variance extracted (AVE) values (0.56–0.73) exceeding the recommended threshold (0.50), indicating that the latent factors captured a substantial proportion of variance. However, comparison of √AVE values with inter-factor correlations showed that the Fornell-Larcker criterion was not met for any construct, indicating limited discriminant validity between the DigiHEALTHQUAL dimensions. Heterotrait-monotrait (HTMT) ratios ranged from 0.74 to 0.88, with no construct pair exceeding the 0.90 threshold, although two pairs fell within the borderline range of 0.85–0.90 (Information & efficiency <> Empathy; Information & efficiency <> Degree of improvement of care services), confirming the pattern of limited but not absent discriminant validity. The observed limited discriminant validity is consistent with the high relatedness of HCSQ dimensions, representing conceptually distinct but closely interconnected concepts^19^. Given the Fornell-Larcker violations and high factor correlations, a higher-order and a unidimensional model were tested.

A higher-order model with HCSQ as an overarching second-order factor was evaluated due to the dominant factor found in the EFA and limited discriminant validity of the first-order model. Second-order loadings were strong (0.80–0.94), indicating substantial shared variance across the four dimensions. At the same time a residual variance remains indicating that the first-order factors are not redundant. A unidimensional model was additionally tested but showed weaker and more heterogeneous factor loadings (0.58–0.84) and lower explained item variance (0.34–0.71), supporting the multidimensional structure over a single-factor solution.

### Confirmatory factor analysis and refinement of the theory-derived model

**Fig. 2:**
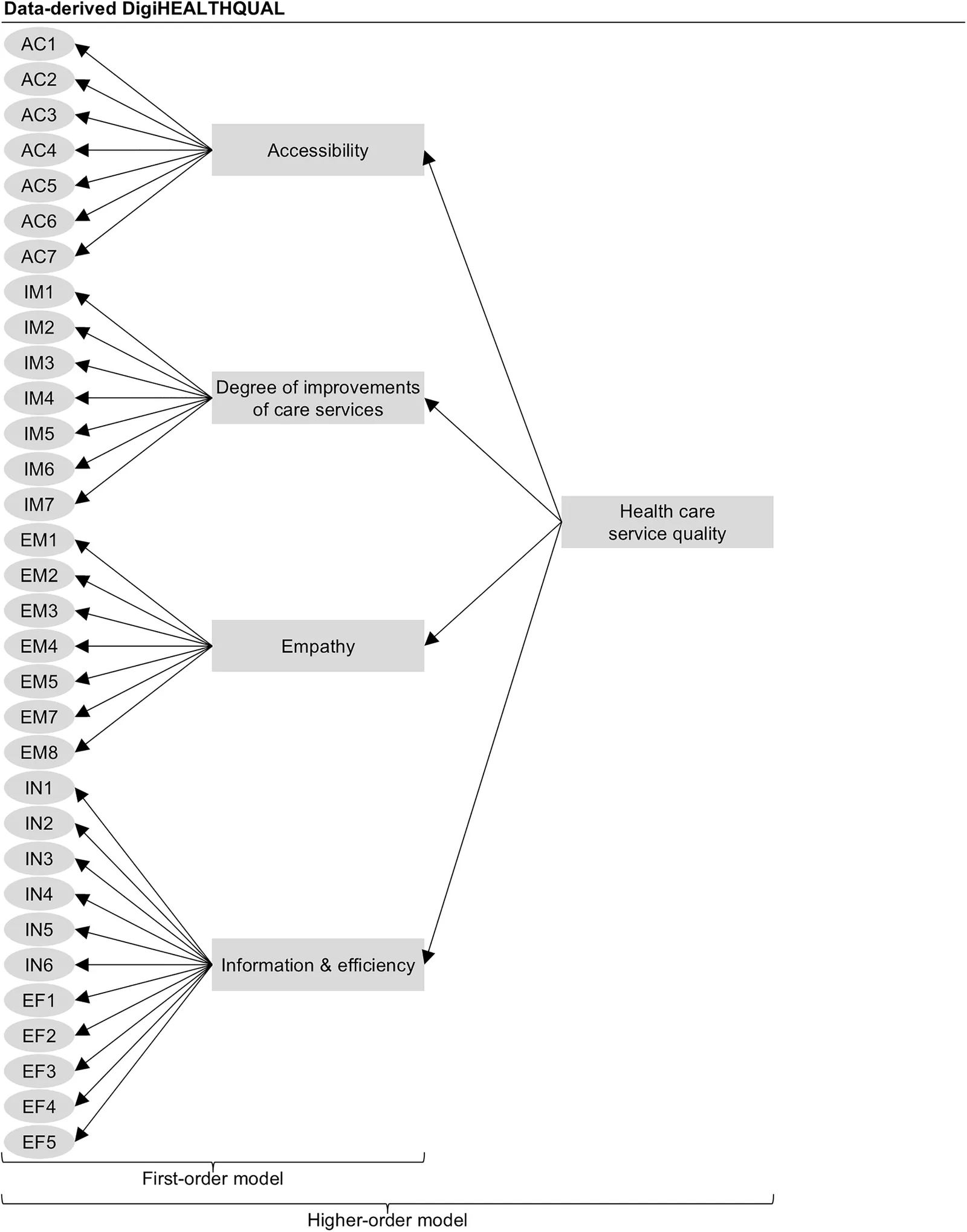
Theory-derived DigiHEALTHQUAL models evaluated using confirmatory factor analysis. The theory-derived DigiHEALTHQUAL instrument comprises 51 items across eight dimensions: general satisfaction (2 items), accessibility (7 items), degree of improvement of care services (7 items), tangibles (5 items), empathy (9 items), information (6 items), efficiency (8 items), and safety (7 items). Confirmatory factor analysis assessed the full eight-dimension model, a refined model excluding the general satisfaction dimension as well as an unidimensional model with all items loading on healthcare service quality.

The theory-derived eight-factor DigiHEALTHQUAL model was evaluated using a final sample of *N* = 843 (Fig. 2). Inspection of the CFA results revealed that one item measuring general satisfaction (GS2) showed a negligible standardized factor loading (0.12) and minimal explained variance (0.02), indicating that it did not function as a reliable indicator of the intended latent factor. Given that the general satisfaction dimension contains only two items, the poor performance of GS2 undermined factor reliability. In addition, general satisfaction has shown high associations with multiple HCSQ dimensions in line with previous studies^25,26^, suggesting empirical redundancy. Consequently, these findings provided justification for excluding the general satisfaction dimension from subsequent analyses.

CFA was repeated for a reduced seven-factor model excluding the general satisfaction dimension (Fig. 2). All retained items demonstrated strong standardized factor loadings (0.70-0.89) and squared multiple correlations (0.49-0.79), indicating a strong measurement quality across all seven dimensions of the refined model (Supplementary Table 4).

The results of the reliability and validity assessment are comparable to the previous CFA of the data-derived model: Latent factor correlations were high (0.77–0.95; Table 2). Internal consistency estimates were high for all constructs indicating potential item redundancy. CR values were acceptable and identical to the respective Cronbach’s α. All constructs demonstrated adequate convergent validity (AVE = 0.56–0.75). The Fornell-Larcker criterion was not met for any construct, indicating a lack of discriminant validity. HTMT ratios exceeded the threshold of 0.90 for three cases (Empathy <> Safety, Information <> Empathy, Efficiency <> Safety), 10 cases were in the borderline area of 0.85–0.90.

**Table 2:** Confirmatory factor analysis: Quality assessment of the refined theory-derived DigiHEALTHQUAL model

Construct	Accessibility	Efficiency	Empathy	Degree of improvement of care services	Information	Safety	Tangibles
Accessibility	—						
Efficiency	0.77	—					
Empathy	0.77	0.89	—				
Degree of improvement of care services	0.77	0.87	0.85	—			
Information	0.71	0.91	0.86	0.86	—		
Safety	0.75	0.95	0.91	0.85	0.88	—	
Tangibles	0.84	0.88	0.87	0.88	0.80	0.86	—
Cronbach	0.90	0.94	0.96	0.94	0.95	0.94	0.91
Composite reliability	0.90	0.94	0.96	0.94	0.95	0.95	0.90
Average inter-item correlation	0.56	0.64	0.71	0.68	0.75	0.71	0.66
Corrected item-total correlation (range)	0.66–0.73	0.72–0.82	0.77–0.86	0.78–0.82	0.80–0.86	0.76–0.85	0.71–0.80
AVE	0.56	0.64	0.71	0.68	0.75	0.71	0.66
√AVE	0.75	0.80	0.84	0.83	0.86	0.84	0.81
HTMT (range)	0.72–0.84	0.78–0.95	0.78–0.91	0.77–0.88	0.72–0.91	0.76–0.95	0.80–0.88

Inter-factor correlation matrix of the retained seven DigiHEALTHQUAL factors; reliability assessment using Cronbach’s α, composite reliability (CR), average inter-item correlation and corrected item-total correlation, and convergent and discriminant validity assessment based on average variance extracted (AVE), √AVE, and heterotrait-monotrait ratios (HTMT).

Similar to the previous CFA, a unidimensional model was tested in which all 49 items loaded onto one latent HCSQ factor. Standardized factor loadings were relatively weaker and more heterogeneous compared to the seven-factor model (0.59–0.84), and squared multiple correlations were comparably lower (0.35–0.70), indicating that a single general factor accounts for considerably less item variance than the multidimensional structure.

### Comparison of theory- and data-derived model

Model fit was compared between the CFAs of the refined theory-derived seven-factor DigiHEALTHQUAL model and both variants (first-order and higher-order) of the data-derived four-factor model as well as the unidimensional models for each set of items (Table 3). The first three models demonstrated good overall fit, with excellent Comparative Fit Index (CFI) and Tucker-Lewis Index (TLI). The theory-derived model showed slightly more favorable absolute fit indices, whereas the data-derived models exhibited greater statistical parsimony, as indicated by lower Akaike Information Criterion (AIC) values. In contrast, both unidimensional models showed substantially worse fit across all indices, with CFI and TLI dropping below 0.90 and Root Mean Square Error of Approximation (RMSEA) approximately doubling compared to the multidimensional models, providing empirical evidence against a strictly unidimensional structure.

**Table 3:** Comparative model fit of assessed DigiHEALTHQUAL models

Model	χ²/df	CFI	TLI	RMSEA	90% CI	PCLOSE	AIC
Theory-derived seven-factor DigiHEALTHQUAL	2.77	0.95	0.95	0.046	0.044–0.048	1.000	3298
Data-derived, first-order four-factor DigiHEALTHQUAL	3.48	0.95	0.95	0.054	0.051–0.057	0.007	1736
Data-derived, higher-order four-factor DigiHEALTHQUAL	3.51	0.95	0.95	0.055	0.052–0.058	0.004	2142
Theory-derived, unidimensional DigiHEALTHQUAL	6.26	0.85	0.84	0.079	0.077–0.081	0.000	7251
Data-derived, unidimensional DigiHEALTHQUAL	9.31	0.83	0.84	0.099	0.097–0.102	0.000	4448

The model fit of three DigiHEALTHQUAL models (theory-derived seven-factor vs. first-order/higher-order data-derived four-factor model) and the unidimensional models for each set of items was assessed after confirmatory factor analysis using χ²/df (acceptable ≤ 3.00), Comparative Fit Index (CFI ≥ 0.90 acceptable, ≥ 0.95 excellent), Tucker-Lewis Index (TLI ≥ 0.90 acceptable, ≥ 0.95 excellent), Root Mean Square Error of Approximation (RMSEA ≤ 0.10 acceptable, ≤ 0.06 excellent; with lower and upper bound 90% confidence interval and with PCLOSE ≥ 0.05), and Akaike Information Criterion (AIC).

Despite their better parsimony, the data-derived models reflected a reduced dimensional structure in which several theoretically established domains (i.e. safety and tangibles) did not emerge as distinct first-order constructs. Their exclusion likely reflects restricted variance and ceiling effects in the sample rather than conceptual irrelevance. Excluding these dimensions would reduce the content validity of DigiHEALTHQUAL as a comprehensive measure of HCSQ. Therefore, the refined theory-derived seven-factor model offers a more comprehensive and conceptually robust framework for DigiHEALTHQUAL and will be retained for subsequent applications.

## Discussion

This study aimed to quantitatively validate DigiHEALTHQUAL as an instrument to assess the effects of DHS on HCSQ in outpatient care and to compare a theory-derived model from our previous study^24^ with the data-derived DigiHEALTHQUAL. Using a large patient sample across four relevant DHS, several key findings emerged from the analysis of multiple variants of DigiHEALTHQUAL:

First, across all tested models, patients perceived DHS effects as highly interrelated dimensions of HCSQ rather than as clearly separable constructs. This was reflected in high inter-factor correlations, violations of discriminant validity criteria including Fornell-Larcker and HTMT, and the substantially worse fit of unidimensional models compared to all multidimensional structures.

Second, the original theory-derived eight-dimension model required refinement. The general satisfaction dimension did not function as an independent first-order construct in CFA. After its removal, the refined seven-factor model demonstrated good to excellent overall model fit, although discriminant validity between HCSQ dimensions remained limited.

Third, EFA yielded a more parsimonious first-order four-factor model characterized by a dominant process-related factor combining information and efficiency, alongside three smaller factors (accessibility, empathy, and degree of improvement of care services). This model highlights the central role of process-related DHS effects on HCSQ and indicates that the theoretically distinct dimensions are empirically closely linked.

Fourth, safety and tangibles did not emerge as distinct factors in EFA, likely due to restricted variance and ceiling effects. Rather than indicating irrelevance, this suggests that patients may perceive these dimensions as baseline expectations.

Finally, a higher-order four-factor model provided empirical support for HCSQ as an overarching construct explaining a substantial proportion of variance in the first-order dimensions. These findings support DigiHEALTHQUAL as a valid measure of DHS effects on HCSQ and underscore the integrated and hierarchical nature of HCSQ perceptions. Importantly, the empirical superiority of multidimensional models over a unidimensional alternative provides justification for retaining distinct dimensions despite their high intercorrelations.

Overall, these findings suggest that the strong integration of non-clinical DHS effects at the level of HCSQ may help explain the limited comparability observed in prior DHS evaluations, especially when narrow or non-validated instruments are used. By capturing non-clinical effects within an integrated HCSQ framework, DigiHEALTHQUAL addresses a key limitation of existing evaluation approaches by making HCSQ measurable in a way that reflects this interconnectedness.

The finding that general satisfaction did not emerge as a distinct first-order dimension is consistent with prior HCSQ research, which has repeatedly shown that global satisfaction measures overlap conceptually with HCSQ dimensions^27,28^. Accordingly, satisfaction and HCSQ are often treated as interrelated but distinct constructs, as high satisfaction does not necessarily imply high HCSQ^26,29^.

The dominance of a process-related factor combining information and efficiency aligns with literature associating DHS with improved organization and processes. Previous studies indicate that patients can benefit from this process-related impact of DHS, for instance through improved access to information^30^, reduced waiting times^31^, and streamlined organizational processes^4^. Yet, the empirical convergence of information and efficiency in the present study suggests that these aspects are experienced as an integrated process dimension rather than as separable components of HCSQ.

In contrast, the absence of tangibles and safety as empirically distinct factors diverges from findings in prior HCSQ research, where these dimensions often demonstrate greater variability and discriminative power, particularly in inpatient settings^19,32,33^. This discrepancy may be attributable to the outpatient context and the characteristics of the present sample. In more heterogeneous or international samples, variation in physical infrastructure and safety perceptions may be greater. Moreover, the perceived importance of safety is likely more pronounced in inpatient settings, where clinical risks are higher due to the type of medical interventions^34–36^. In outpatient care, by contrast, tangibles and safety may function as baseline expectations or “hygiene factors” rather than as attributes that differentiate perceived HCSQ. The observed ceiling effects and restricted variance support this interpretation, suggesting that these dimensions become important primarily when expectations are violated rather than when they are met^37,38^. Additionally, the health care sector is comparably highly regulated leading to a relatively high degree of standardization, particularly with regard to safety requirements. This uniformity likely constrains observable variation and contributes to the limited discriminative capacity of affected HCSQ dimensions.

This is the first study to validate a HCSQ-based instrument for DHS assessment in Germany. A major strength of this study is the broad scope spanning multiple DHS with one of the largest patient samples studied for this purpose in Germany, which enabled robust psychometric analyses and an explicit comparison of theory-derived and data-derived model structures. The study builds on a theoretically grounded instrument development process and applies a rigorous validation strategy, including EFA and CFA as well as higher-order modeling. Extensive data quality assurance procedures and transparent reporting further strengthen the credibility and reproducibility of the findings.

Several limitations should be considered. First, the use of a voluntary, online survey and convenience sampling may limit generalizability and likely introduces a bias towards participants with higher digital affinity. Compared to the general population in Germany, the sample shows some deviations in sociodemographic characteristics, including a higher proportion of male participants and differences in age distribution, with younger and older age groups being slightly underrepresented. Minor deviations were also observed regarding place of residence, with smaller and medium-sized places of residence being relatively overrepresented. No relevant deviations were observed with regard to health insurance status. However, the sample still reflects a broad range of patient characteristics, and such deviations are common in digitally recruited samples. Given the study’s focus on the validation of a measurement instrument rather than population-level estimates, these differences are not expected to substantially affect the interpretation of the factor structure. Second, the study was conducted exclusively in Germany; therefore, the findings may not directly transfer to health care systems with different regulatory, organizational, or cultural contexts. Third, respondents evaluated only one DHS, which precluded within-person comparisons across multiple DHS or assessment of cumulative digitalization effects. Fourth, listwise deletion was applied in factor analyses due to low overall missingness and software constraints. Although this approach is unlikely to have materially biased the results, it may have reduced the effective sample size for analyses. Fifth, the consistently high internal consistency estimates and the lack of discriminant validity across models indicate potential item redundancy and strong interdependence among HCSQ dimensions. However, this pattern should not be interpreted solely as a psychometric limitation, as it reflects the empirical reality that patients experience multiple aspects of HCSQ as closely intertwined, supporting the conceptualization of HCSQ as a hierarchical and integrated construct. To further examine these patterns, average inter-item correlations, corrected item-total correlations, and HTMT ratios were analyzed. While average inter-item correlations exceeded the ideal range suggesting narrow construct coverage and potential for further future item reduction, uniformly strong corrected item-total correlations indicated that no individual item performed poorly, making it difficult to identify specific items for removal without further empirical guidance. Sixth, although the item set was reduced, the resulting instrument may still be relatively lengthy, potentially limiting its feasibility for routine use in time-constrained outpatient care settings and highlighting the need for further scale refinement.

Finally, although DigiHEALTHQUAL was designed for multi-stakeholder assessment, the present validation focused exclusively on patients. Validation studies including health care professionals are therefore necessary to fully establish the instrument’s applicability across stakeholder groups.

From a practical perspective, the refined theory-derived DigiHEALTHQUAL model appears suitable for comprehensive post-implementation evaluation of DHS effects on HCSQ in outpatient care, preserving conceptual breadth and alignment with established theory. Although the data-derived model demonstrated greater statistical parsimony, its reduced dimensionality would limit content validity by omitting domains relevant for comprehensive HCSQ assessment. At the same time, the data-derived structure provides a useful empirical basis for the future development of a shortened version of DigiHEALTHQUAL.

From a broader evaluation perspective, DigiHEALTHQUAL complements established health technology assessment and implementation frameworks by systematically capturing non-clinical effects that are often underrepresented in evaluations focused on clinical effectiveness. While clinical evidence remains essential, HCSQ dimensions such as accessibility or information provision constitute important components of perceived value in outpatient care. By providing a standardized and theory-based measure of these effects, DigiHEALTHQUAL can serve as a structured input to post-implementation evaluations alongside clinical evidence.

For practice owners, managers, payers, and policymakers, the findings suggest that patients primarily perceive the impact of DHS through process-related improvements, particularly in information provision and organizational efficiency. In contrast, dimensions such as safety and tangibles appear to function as baseline expectations shaped by regulatory standards rather than as differentiating drivers of perceived value. This underscores the importance of considering the effects of DHS on processes and patient interaction, provided that baseline requirements are met.

Importantly, DigiHEALTHQUAL should be understood primarily as a measurement contribution rather than a standalone decision-support tool. Its immediate value lies in enabling standardized and comparable post-implementation assessments of DHS effects on HCSQ. Only with the accumulation of comparative data across multiple DHS and care settings can DigiHEALTHQUAL meaningfully inform prospective decision-making by practice owners, managers, payers, or policymakers. For example, DigiHEALTHQUAL could support decision-making by enabling comparisons between different DHS with regard to their impact on HCSQ dimensions, such as empathy or tangibles. Health care providers may use these insights to prioritize the implementation of solutions that show the greatest perceived benefits. In addition, the instrument can help identify specific areas of improvement within existing DHS, thereby informing targeted optimization efforts.

Future research should build on the higher-order structure supported in this study by testing bifactor models, which would allow estimation of additional indices to further clarify the relative contribution of the general HCSQ factor versus specific subdimensions. Additionally, measurement invariance across DHS types, demographic groups, and levels of digital affinity should be examined. Replication in other countries and health care systems is essential to establish broader generalizability, and dedicated validation studies including health care professionals are particularly needed. In addition, the development and validation of a shortened DigiHEALTHQUAL version may facilitate routine monitoring and large-scale applications.

This study provides the first large-scale quantitative validation of DigiHEALTHQUAL in a patient cohort and demonstrates its applicability for evaluating non-clinical effects of DHS on HCSQ in outpatient care. While the original theory-derived structure required refinement, the retained seven-factor model offers a conceptually robust and comprehensive framework that aligns with both theoretical expectations and empirical evidence. By operationalizing HCSQ as an integrated evaluative construct, DigiHEALTHQUAL addresses a key gap in the assessment of DHS and provides a foundation for more comparable, theory-informed evaluation of DHS in future research and practice.

## Methods

### Study design

**Fig. 3:**
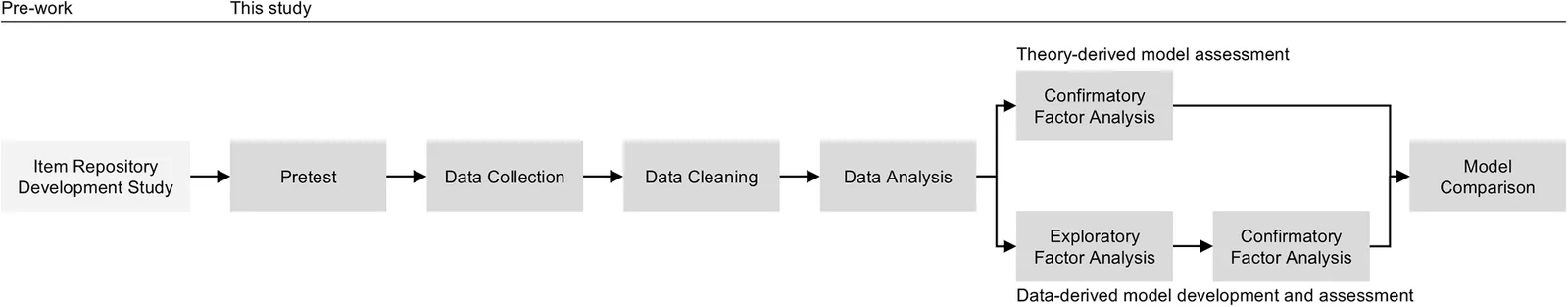
Study design and workflow. The DigiHEALTHQUAL questionnaire was refined through pretesting, followed by data collection and cleaning. Two analytical pathways were applied: validation of the theory-derived model using confirmatory factor analysis, and development of a data-derived model using exploratory factor analysis followed by confirmatory factor analysis.

The present study validates DigiHEALTHQUAL^24^ in a patient cohort across four selected DHS. Two complementary analytical paths were followed: (1) validation of the original theory-derived model using confirmatory factor analysis, and (2) development of a data-derived model using exploratory factor analysis followed by confirmatory factor analysis. The resulting models were compared using model fit indices and assessment of factor interpretability. An overview of the analytical workflow is provided in Fig. 3.

### Ethical considerations

The study protocol was approved by the Ethics Committee of Witten/Herdecke University (S-113/2023, clinical trial number: not applicable) in accordance with German General Data Protection Regulation (DSGVO). All participants received written information on study objectives, procedures, and data handling, and provided written informed consent prior to participation. Participation was voluntary and no incentives were offered. To protect participant privacy, all responses were anonymized using the k-anonymity principle; responses not meeting the k ≥ 5 anonymity criterion were removed before analysis. Data were stored on secure, password-protected servers.

### Selection of use cases

Given the broad range of DHS in outpatient care, use cases were narrowed down to four eligible use cases: online appointment booking, digital anamnesis, video consultation, and electronic health records. The use cases referred to general functionalities rather than specific applications or vendors. Use case selection was conducted jointly by a team of five researchers and health care practitioners. Selection criteria were: (1) importance across the patient journey, and (2) perceived practical relevance for both patients and health care practitioners.

### Instrument development and pretesting

The original DigiHEALTHQUAL instrument was developed in English and translated into German for this study. The translation was reviewed and refined by the research team to ensure clarity and conceptual consistency. Face validity was assessed through a structured pretest following recommendations by Boateng et al.^39^ and Allen et al.^40^. Ten patients reviewed the questionnaire for clarity and comprehensiveness as well as usability. The feedback resulted in selected refinements to introductory text, item wording, and selected response options. No structural or conceptual modifications to the instrument were made.

### Participants and recruitment

The survey was administered in German. Participants were eligible if they: (1) were ≥ 18 years old, (2) resided in Germany, and (3) had experience with at least one of the four selected DHS in the last year. Recruitment followed a convenience sampling strategy and used multiple digital channels without provision of incentives, including participants from prior research, personal networks, social media, and an online panel. The voluntary, open-access survey was conducted via LimeSurvey between January and March 2025^41^.

### Survey structure and measures

The survey started with study information including research purpose, target population, length of survey, and ethics committee approval, and a consent form including details on data security and storage. Participants were presented with short definitions of the four use cases and asked to select the one they had used most. Respondents unfamiliar with all use cases exited the survey automatically. Two follow-up questions assessed frequency and duration of use (5- or 6-point Likert-type scales respectively) followed by the DigiHEALTHQUAL instrument^24^, consisting of 51 items across eight HCSQ dimensions derived from HEALTHQUAL^19^, Outpatient Experience Questionnaire^42,43^, and Patient Satisfaction Questionnaire 18^44^. Accordingly, DigiHEALTHQUAL was administered with reference to one specific use case selected by the respondent. Participants were instructed to rate the extent to which that use case affected the different HCSQ dimensions, enabling standardized comparison across different patient-facing use cases within outpatient care. For usability, two dimensions were presented per page: general satisfaction (2 items) and accessibility (7 items); degree of improvement of care services (7 items) and tangibles (5 items); empathy (9 items) and information (6 items); efficiency (8 items) and safety (7 items). Responses were captured on a 6-point Likert-type scale (“strongly disagree” to “strongly agree”), with an additional “I don’t know/prefer not to answer” option. One attention-check item (“2 + 2 = ?”) was embedded after four DigiHEALTQUAL dimensions. The final section collected demographic characteristics (i.e., gender, size of place of residence, age range), perceived digital affinity, and health insurance status. Survey design and documentation followed the Checklist for Reporting Results of Internet E-Surveys (refer to Suppl. Table 5)^45^. The final questionnaire is provided in the appendix (Suppl. Note 1).

### Data cleaning and missing data handling

Data cleaning followed established recommendations for online survey quality control^46,47^ and was performed in Microsoft Excel^48^. Exclusion criteria included: lack of provided consent, lack of experience with selected use cases, incomplete answers, and duplicate entries (assessed via IP address and demographic patterns). Further quality checks excluded responses with incorrect answers of control questions, straight-lining within at least one survey page, no answers for > 10% of DigiHEALTHQUAL items, completion times within the fastest 5%, or not meeting the k ≥ 5 anonymity criterion. All remaining cases were used for analysis. The reverse-coded item GS2 was recoded prior to analysis.

Overall missingness across DigiHEALTHQUAL items was low (total 1%, range per item 0.2–3.6%). Little’s MCAR test^49^ was conducted using IBM SPSS version 29.0^50^ and results were significant (χ²(8063) = 11033.21, *p* < 0.001), which is common in large-scale datasets and does not necessarily indicate meaningful deviation from randomness.

### Statistical analysis

Although DigiHEALTHQUAL is based on a theoretically derived factor structure, this structure represents a synthesis of multiple existing models and has not been empirically validated in its current form. We therefore conducted an EFA to examine whether the assumed dimensionality is supported in the present empirical context. Subsequently, CFA was used to test both the theory-derived model and the empirically derived factor structure. The comparison between models was based on statistical criteria (e.g., model fit and validity measures) and conceptual interpretability, rather than on the theoretical origin of the model alone.

#### Exploratory factor analysis

Normality was assessed using skewness and kurtosis statistics and visual inspections of histograms and Q-Q plots at item and dimension level. EFA was conducted in SPSS^50^ following the recommendations Watkins et al.^51^ and Costello et al.^52^ by using principal axis factoring of the inter-item correlation matrix with direct oblimin rotation, based on theoretical expectation of correlation between dimensions. Factor retention was based on eigenvalues ≥1 and scree plot inspection^53^. Item reduction followed a predefined structured, iterative process: (1) removal of non-loading items (≤ 0.30), (2) removal of cross-loading items (≥0.30 on ≥ 2 factors with loading difference ≤ 0.20), (3) low-loading items (≤0.40)^54,55^. Items were removed one at a time prioritizing removal of the weakest item at each step, and exploratory factor analysis rerun after each iteration. Sampling adequacy was evaluated using the Bartlett’s test of sphericity (< 0.05 significant)^56^ and Kaiser-Meyer-Olkin statistic (>0.9 marvelous)^57^. Because missingness was minimal and SPSS does not support full information maximum likelihood estimation for EFA, listwise deletion was applied.

#### Confirmatory factor analysis

CFA was performed in AMOS^58^ following the recommendations by Jackson et al.^59^. CFA was conducted for six DigiHEALTHQUAL models: (1) the theory-derived eight-factor model^24^, (2) the theory-derived seven-factor model excluding the dimension general satisfaction, (3) the data-derived four-factor model derived from the previous exploratory factor analysis, (4) a higher-order model of the data-derived four-factor model, (5) an unidimensional model based on the theory-derived set of items, and (6) an unidimensional model based on the data-derived set of items. For all first-order models, all latent factors were allowed to correlate. No correlated residuals were specified, and all measurement errors were assumed to be independent. Model identification was achieved by fixing latent variance to 1. For the higher-order model, covariances among first-order factors were not freely estimated but captured via the second-order factor. Model identification was achieved by fixing latent variance and one secondary-order loading to 1. CFA was performed using maximum likelihood estimation due to potential non-normality and the large sample size. Missing data were handled using listwise deletion due to low item-level missingness. Model quality was compared using: χ²/df (acceptable ≤ 3.00)^60^, CFI ( ≥0.90 acceptable, ≥ 0.95 excellent)^55^, TLI ( ≥ 0.90 acceptable, ≥0.95 excellent)^55^, Root Mean Square Error of Approximation (RMSEA ≤0.10 acceptable, ≤0.06 excellent; lower and upper bound 90% confidence interval and with PCLOSE ≥ 0.05)^55,61^, and AIC.

#### Reliability and validity assessment

Reliability and validity were inspected at the factor and item level. Internal consistency was evaluated using Cronbach’s α, average inter-item correlation, corrected item-total correlation, and CR. Cronbach’s α ≥ 0.70 was considered acceptable, with ≥0.80 indicating good and ≥ 0.90 excellent reliability^62,63^. Average inter-item correlation was considered acceptable with ≥ 0.15 and ≤ 0.50^55^. Corrected item-total correlation was considered acceptable with ≥ 0.30^53^. CR was calculated from standardized factor loadings and error variances (≥0.70 acceptable)^64^. Convergent validity was assessed using AVE ( ≥0.50 acceptable). Discriminant validity was evaluated using the Fornell-Larcker criterion by comparing √AVE for each construct with its inter-construct correlations^65^ and calculating HTMT with ≤ 0.85 as good and ≤0.90 as acceptable^66^.

## Supplementary information

Supplementary information

## Data Availability

The datasets generated and/or analyzed during the current study are not publicly available due to privacy and ethical restrictions associated with participant-level data but are available from the corresponding author on reasonable request.
