# Supplementary material for: Validating DigiHEALTHQUAL for measuring digital health effects on service quality in outpatient care in Germany
Source: NPJ Digit Med. 2026 Oct 2;9:751. doi: 10.1038/s41746-026-03302-0 (PMC13633258; doi:10.1038/s41746-026-03302-0)
Supplement: Supplementary file 1 [file 41746_2026_3302_MOESM1_ESM.pdf]

Supplementary Figure 1: Data cleaning and sample selection.

Of 3725 collected responses, 1454 met eligibility criteria (informed consent, experience with relevant digital use cases, complete survey, no duplicate entries). After applying data quality and privacy filters, 1085 responses remained for analysis (control questions passed, no straight-lining, >10% missing responses, exclusion of fastest 5%, k≥5 anonymity).

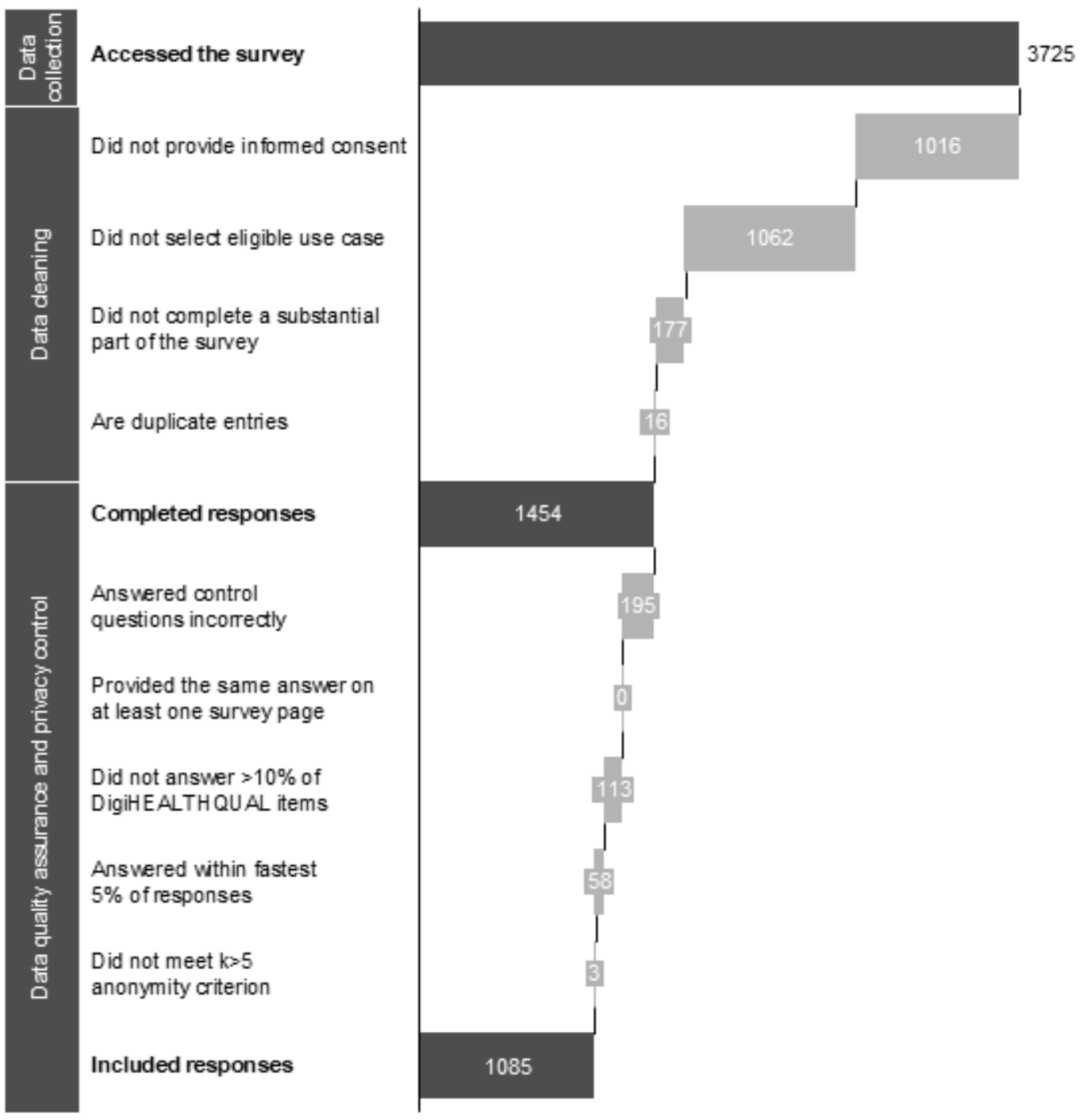

Supplementary Figure 2: Characteristics of survey participants.

Participants were predominately male (662/1085, 61%), aged 55-65 years (264/1085, 24%), most commonly lived in places of residence with 20001-100000 inhabitants (300/1085, 28%), were insured under statutory health insurance (841/1085, 78%) and reported moderate to high digital affinity (488/1085, 45%); percentages may not sum to 100% due to rounding.

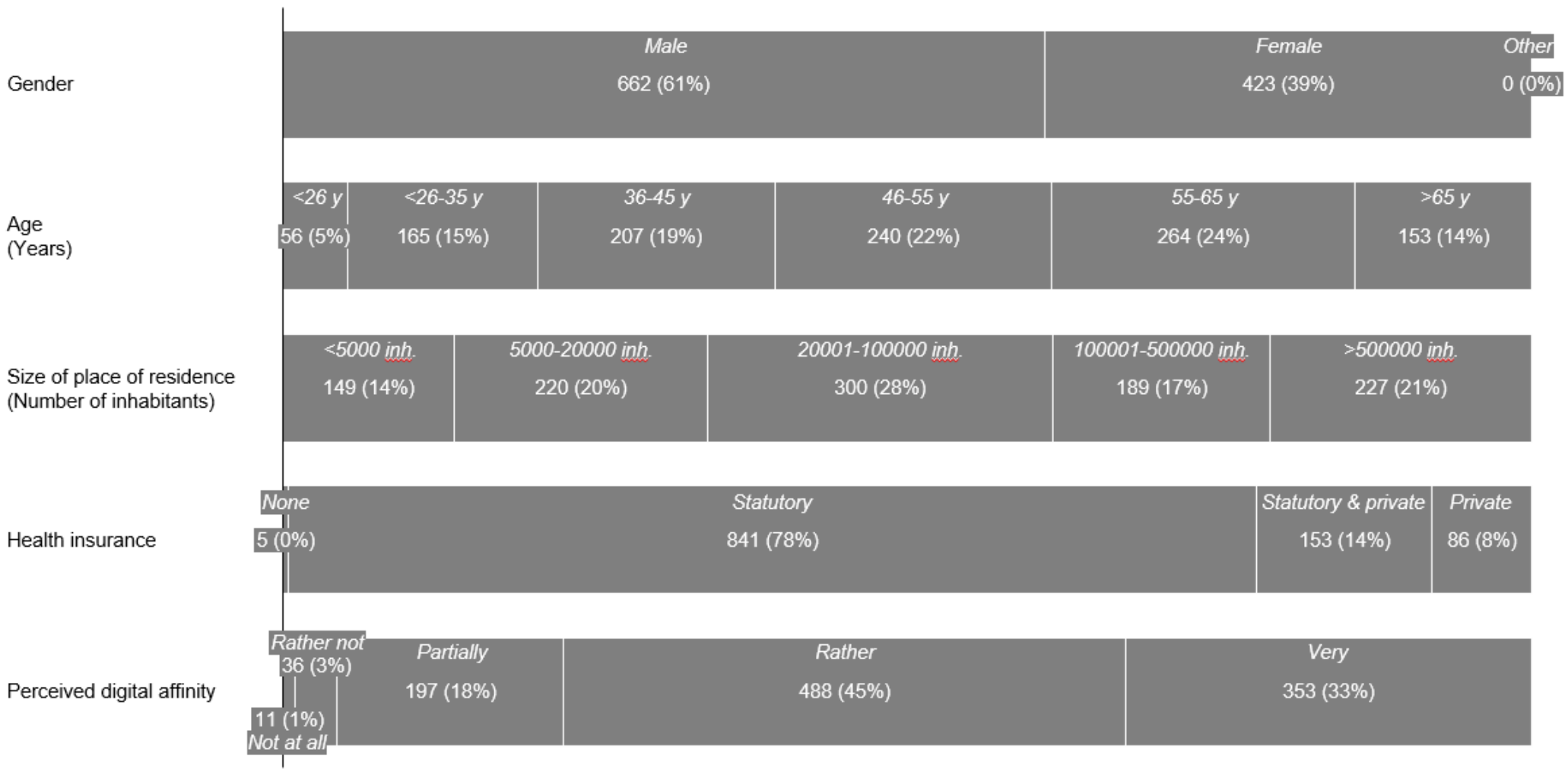

Supplementary Table 1: Results of the exploratory factor analysis of the data-derived DigiHEALTHQUAL model.

Factor	Item	Factor loadings				Eigenvalues	% of variance
		1	2	3	4		
Information & efficiency	IN5	.86				18.96	58.25
	IN2	.85					
	EF4	.84					
	IN4	.83					
	IN6	.81					
	EF1	.81					
	IN1	.78					
	IN3	.70					
	EF2	.65					
	EF5	.55					
	EF3	.52					
Accessibility	AC3		.83			1.78	4.41
	AC2		.78				
	AC6		.72				
	AC5		.68				
	AC1		.61				
	AC4		.54				
	AC7		.51				
Empathy	EM3			.81		1.13	2.63
	EM4			.78			
	EM5			.78			
	EM2			.78			
	EM8			.77			
	EM1			.74			
	EM7			.73			
Degree of improvement of care services	IM7				.88	1.01	2.21
	IM4				.86		
	IM3				.83		
	IM6				.56		
	IM2				.56		
	IM5				.56		
	IM1				.54		

Supplementary Table 2: Correlation matrix of the data-derived DigiHEALTHQUAL model.

Construct	Accessibility (2)	Degree of improvement of care services (4)	Empathy (3)	Information & efficiency (1)
Accessibility	-			
Degree of improvements of care services	.66	-		
Empathy	.67	.76	-	
Information & efficiency	.64	.80	.83	-

Supplementary Table 3: Results of the confirmatory factor analysis of the data-derived DigiHEALTHQUAL model.

Table 3. Results of the confirmatory factor analysis of the data-derived DigiHEALTHQUAL model.
Standardized factor loadings and squared multiple correlations (R^2) for each of the 32 items loading on its respective latent factor (accessibility, degree of improvement of care services, empathy, information & efficiency).

Factor	Item	Standardized factor loadings	Squared multiple correlations (R^2)
Accessibility	AC1	.73	.53
	AC2	.78	.61
	AC3	.76	.58
	AC4	.74	.54
	AC5	.74	.54
	AC6	.77	.60
	AC7	.71	.50
Degree of improvement of care services	IM1	.84	.71
	IM2	.81	.66
	IM3	.84	.71
	IM4	.83	.69
	IM5	.81	.66
	IM6	.82	.67
	IM7	.82	.67
Empathy	EM1	.81	.66
	EM2	.87	.76
	EM3	.88	.78
	EM4	.86	.74
	EM5	.84	.70
	EM7	.86	.74
	EM8	.87	.75
Information & efficiency	IN1	.83	.68
	IN2	.85	.73
	IN3	.83	.69
	IN4	.86	.75
	IN5	.88	.77
	IN6	.87	.76
	EF1	.79	.63
	EF2	.84	.70
	EF3	.80	.64
	EF4	.81	.65
	EF5	.78	.61

Supplementary Table 4: Results of the confirmatory factor analysis of the refined theory-derived DigiHEALTHQUAL model.

Factor	Item	Standardized factor loadings	Squared multiple correlations (R^2)
Accessibility	AC1	0.73	0.53
	AC2	0.78	0.61
	AC3	0.77	0.59
	AC4	0.73	0.54
	AC5	0.74	0.55
	AC6	0.78	0.60
	AC7	0.70	0.49
Efficiency	EF1	0.79	0.63
	EF2	0.85	0.72
	EF3	0.83	0.69
	EF4	0.81	0.66
	EF5	0.81	0.65
	EF6	0.83	0.69
	EF7	0.75	0.56
	EF8	0.74	0.55
Empathy	EM1	0.82	0.67
	EM2	0.86	0.75
	EM3	0.88	0.78
	EM4	0.86	0.74
	EM5	0.84	0.71
	EM6	0.79	0.63
	EM7	0.85	0.73
	EM8	0.86	0.74
	EM9	0.82	0.67
Degree of improvement of care services	IM1	0.85	0.72
	IM2	0.82	0.66
	IM3	0.84	0.70
	IM4	0.83	0.69
	IM5	0.81	0.66
	IM6	0.82	0.67
	IM7	0.81	0.66
Information	IN1	0.83	0.69
	IN2	0.86	0.75
	IN3	0.84	0.70
	IN4	0.88	0.77
	IN5	0.89	0.79
	IN6	0.89	0.79
Safety	SA1	0.86	0.74
	SA2	0.84	0.70
	SA3	0.87	0.75
	SA4	0.88	0.77
	SA5	0.78	0.60
	SA6	0.87	0.76
	SA7	0.82	0.66
Tangibles	TA1	0.73	0.54
	TA2	0.83	0.68
	TA3	0.81	0.65
	TA4	0.85	0.73
	TA5	0.82	0.68

Supplementary Table 5: Completed Checklist for Reporting Results of Internet E-Surveys (CHERRIES).

ITEM CATEGORY	ITEM	EXPLANATION	REPORTED IN SECTION
**DESIGN**			
Describe survey design	1	Describe target population, sample frame. Is the sample a convenience sample? (In “open” surveys this is most likely.)	see ‘Participants and Recruitment’
**IRB (Institutional Review Board) APPROVAL AND INFORMED CONSENT PROCESS**			
IRB approval	2	Mention whether the study has been approved by an IRB.	see ‘Ethical Considerations’
Informed consent	3	Describe the informed consent process. Where were the participants told the length of time of the survey, which data were stored and where and for how long, who the investigator was, and the purpose of the study?	see ‘Survey Structure and Measures’
Data protection	4	If any personal information was collected or stored, describe what mechanisms were used to protect unauthorized access.	see ‘Ethical Considerations’
**DEVELOPMENT AND PRE-TESTING**			
Development and testing	5	State how the survey was developed, including whether the usability and technical functionality of the electronic questionnaire had been tested before fielding the questionnaire.	see ‘Instrument Development and Pretesting’ and ‘Survey Structure and Measures’
**RECRUITMENT PROCESS AND DESCRIPTION OF THE SAMPLE HAVING ACCESS TO THE QUESTIONNAIRE**			
Open survey versus closed survey	6	An “open survey” is a survey open for each visitor of a site, while a closed survey is only open to a sample which the investigator knows (password-protected survey).	see ‘Participants and Recruitment’
Contact mode	7	Indicate whether or not the initial contact with the potential participants was made on the Internet. (Investigators may also send out questionnaires by mail and allow for Web-based data entry.)	see ‘Participants and Recruitment’
Advertising the survey	8	How/where was the survey announced or advertised? Some examples are offline media (newspapers), or online (mailing lists – If yes, which	see ‘Participant and Recruitment’<br>The communication used for recruitment was slightly adjusted for each audience and channel. Overall, we provided the different

ITEM CATEGORY	ITEM	EXPLANATION	REPORTED IN SECTION
		ones?) or banner ads (Where were these banner ads posted and what did they look like?). It is important to know the wording of the announcement as it will heavily influence who chooses to participate. Ideally the survey announcement should be published as an appendix.	channels with an email or post, a digital flyer containing the most relevant information about the survey, and the respective survey link.<br>The email or post included the following message (translated from German to English): "Dear [name], would you like to know how digitalization is changing primary and specialist care practices, and how patients and healthcare professionals perceive these changes? We are investigating this question at the Chair of Health Informatics at Witten/Herdecke University. Help us better understand the impact of digital tools on service quality in outpatient practices by taking part in our 15-minute anonymous survey. Feel free to share the link within your network!"<br>The digital flyer included the following message (translated from German to English): "What impact does digitalization have in your medical practice? Participants wanted for a survey, Study conducted by the Chair of Healthcare Informatics at Witten/Herdecke University. What is the survey about? Evaluation of the impact of digital tools on service quality in outpatient practices in Germany, from the patients' perspective (ethics approval S--113/2023). Participate in the survey now! Who can take part in the survey? Patients from general and specialist outpatient practices in Germany who have experience using at least one of the following applications: Online appointment booking, digital anamneses questionnaires, video consultations, electronic patient records. How is the survey conducted? The survey is anonymous, takes approximately 15 minutes, and includes questions on service quality, use of digital tools, and sociodemographic factors."
**SURVEY ADMINISTRATION**			
Web/E-Mail	9	State the type of e-survey (e.g., one posted on a Web site, or one sent out through e-mail). If it is an e-mail survey, were the responses entered manually into a database, or was there an automatic method for capturing responses?	see 'Participants and Recruitment'
Context	10	Describe the Web site (for mailing list/newsgroup) in which the survey was posted. What is the Web site	n/a

ITEM CATEGORY	ITEM	EXPLANATION	REPORTED IN SECTION
		about, who is visiting it, what are visitors normally looking for? Discuss to what degree the content of the Web site could pre-select the sample or influence the results. For example, a survey about vaccination on an anti-immunization Web site will have different results from a Web survey conducted on a government Web site	
Mandatory/voluntary	11	Was it a mandatory survey to be filled in by every visitor who wanted to enter the Web site, or was it a voluntary survey?	see 'Participants and Recruitment'
Incentives	12	Were any incentives offered (e.g., monetary, prizes, or non-monetary incentives such as an offer to provide the survey results)?	see 'Ethical Considerations'
Time/date	13	In what timeframe were the data collected?	see 'Participants and Recruitment'
Randomization of items or questionnaires	14	To prevent biases items can be randomized or alternated.	The overall structure of the survey was the same for all respondents. The order of items within each DigiHEALTHQUAL subdimension was randomized.
Adaptive questioning	15	Use adaptive questioning (certain items, or only conditionally displayed based on responses to other items) to reduce number and complexity of the questions.	Adaptive questioning was neither feasible nor required for the chosen questionnaire design.
Number of items	16	What was the number of questionnaire items per page? The number of items is an important factor for the completion rate.	see 'Survey Structure and Measures'<br>Our survey containing in total 66 items.
Number of screens (pages)	17	Over how many pages was the questionnaire distributed? The number of items is an important factor for the completion rate.	The questionnaire was distributed across ten pages. The first page presented a welcome message and study description, followed by a second page providing information on data protection and data processing and obtaining informed consent. The third page covered use case selection. Detailed questions related to the selected use case were presented on the fourth page. To reduce respondent burden and limit the number of items per page, the DigiHEALTHQUAL instrument was divided across four consecutive pages (pages five to eight), each displaying two dimensions of the instrument. Sociodemographic characteristics were collected on the ninth page. The final, tenth page displayed a farewell message and concluded the survey.

ITEM CATEGORY	ITEM	EXPLANATION	REPORTED IN SECTION
Completeness check	18	It is technically possible to do consistency or completeness checks before the questionnaire is submitted. Was this done, and if “yes”, how (usually JAVAScript)? An alternative is to check for completeness after the questionnaire has been submitted (and highlight mandatory items). If this has been done, it should be reported. All items should provide a non-response option such as “not applicable” or “rather not say”, and selection of one response option should be enforced.	We utilized forced responses for the first three pages and soft-forced-response (i.e., reminders to provide complete answers) for the latter. Starting on page 4, all items included a non-response option (“I don’t know/prefer not to answer”). Forced response was also applied for the sociodemographic questions.
Review step	19	State whether respondents were able to review and change their answers (e.g., through a Back button or a Review step which displays a summary of the responses and asks the respondents if they are correct).	We decided not to include a "back" button for respondents to review and change their responses to counter a potential social desirability effect that might cause them to change their responses after completing subsequent questions.
**RESPONSE RATES**			
Unique site visitors	20	If you provide view rates or participation rates, you need to define how you determined a unique visitor. There are different techniques available, based on IP addresses or cookies or both.	see ‘Data Cleaning and Missing Data Handling’
View rate (Ratio of unique survey visitors/unique site visitors)	21	Requires counting unique visitors to the first page of the survey, divided by the number of unique site visitors (not page views!). It is not unusual to have view rates of less than 0.1 % if the survey is voluntary.	see ‘Sample Characteristics’
Participation rate (ratio of those who agreed to participate/unique first survey page visitors)	22	Count the unique number of people who filled in the first survey page (or agreed to participate, for example by checking a checkbox), divided by visitors who visit the first page of the survey (or the informed consents page, if present). This can also be called “recruitment” rate.	see ‘Sample Characteristics’
Completion rate (ratio of users who finished the survey/users who agreed to participate)	23	The number of people submitting the last questionnaire page, divided by the number of people who agreed to participate (or submitted the first survey page). This is only relevant if there is a separate “informed consent” page or if the survey	see ‘Sample Characteristics’

ITEM CATEGORY	ITEM	EXPLANATION	REPORTED IN SECTION
		goes over several pages. This is a measure for attrition. Note that "completion" can involve leaving questionnaire items blank. This is not a measure for how completely questionnaires were filled in. (If you need a measure for this, use the word "completeness rate".)	
**PREVETING MULTIPLE ENTRIES FROM THE SAME INDIVIDUAL**			
Cookies used	24	Indicate whether cookies were used to assign a unique user identifier to each client computer. If so, mention the page on which the cookie was set and read, and how long the cookie was valid. Were duplicate entries avoided by preventing users access to the survey twice; or were duplicate database entries having the same user ID eliminated before analysis? In the latter case, which entries were kept for analysis (e.g., the first entry or the most recent)?	We decided not to use cookies to prevent multiple entries from the same individual, as participants living in the same household might share an IP address. Thus, we would automatically exclude them from participating in the survey.
IP check	25	Indicate whether the IP address of the client computer was used to identify potential duplicate entries from the same user. If so, mention the period of time for which no two entries from the same IP address were allowed (e.g., 24 hours). Were duplicate entries avoided by preventing users with the same IP address access to the survey twice; or were duplicate database entries having the same IP address within a given period of time eliminated before analysis? If the latter, which entries were kept for analysis (e.g., the first entry or the most recent)?	see 'Data Cleaning and Missing Data Handling<br>We checked the anonymized IP addresses of participants to identify duplicate entries from the same individual. However, we only excluded IP address duplicates with identical demographic data to ensure that we would allow participants from the same household sharing an IP address to participate in the survey. We kept the first complete entry for the cases with identical IP addresses and demographic data.
Log file analysis	26	Indicate whether other techniques to analyze the log file for identification of multiple entries were used. If so, please describe.	n/a
Registration	27	In "closed" (non-open) surveys, users need to login first and it is easier to prevent duplicate entries from the same user. Describe how this was done. For example, was the survey never displayed a second	n/a

ITEM CATEGORY	ITEM	EXPLANATION	REPORTED IN SECTION
		time once the user had filled it in, or was the username stored together with the survey results and later eliminated? If the latter, which entries were kept for analysis (e.g., the first entry or the most recent)?	
**ANALYSIS**			
Handling of incomplete responses	28	Were only completed questionnaires analyzed? Were questionnaires which terminated early (where, for example, users did not go through all questionnaire pages) also analyzed?	see 'Data Cleaning and Missing Data Handling'
Questionnaires submitted with atypical timestamp	29	Some investigators may measure the time people needed to fill in a questionnaire and exclude questionnaires that were submitted too soon. Specify the timeframe that was used as a cut-off point and describe how this point was determined.	see 'Data Cleaning and Missing Data Handling'
Statistical correction	30	Indicate whether any methods such as weighting of items or propensity scores have been used to adjust for the non-representative sample; if so, please describe the methods.	n/a

Supplementary Note 1: Translated Questionnaire.

Disclaimer: The questionnaire was distributed in German. Item IDs were not shown to survey participants.

Welcome and Introduction

Dear prospective participant,

Thank you very much for your interest in this survey.

In this survey, we aim to investigate your opinion on the effects of digitalization on service quality in outpatient practices. For this purpose, we have developed a questionnaire that we would now like to validate while simultaneously gaining initial insights.
Completing the survey will take approximately 10 minutes. In the interest of scientific knowledge generation, we kindly ask you to complete the questionnaire in full.
The Ethics Committee of Witten/Herdecke University has positively evaluated this study under application number S-113/2023. On the following pages, you will receive all necessary information regarding your participation in this study, data processing, and your rights as a participant. Subsequently, we will ask you to provide your consent to participate.

Thank you very much for your support.

Informed Consent

I have been informed that, as part of this online survey, personal data of participants are collected and further processed in anonymized, aggregated form.

I have been informed about the purpose, scope, legal basis, and duration of data storage, as well as about the rights of study participants with regard to their personal data. I have received a corresponding information sheet.

I am aware that participation is voluntary and that I may withdraw my consent at any time, without providing reasons and without any personal disadvantage, either in writing or verbally.

Show Consent Statement

☐ I agree to participate in the above-mentioned study and consent to the associated and known processing of my personal data.

Selection of Digital Health Solutions

In this survey, we ask you to select one digital health solution that you have already used in one of your outpatient practices (general practitioner or specialist). If you have used multiple solutions, please freely choose one solution. We recommend selecting the solution you have used most frequently. The digital health solutions considered in this study are:

- Online appointment booking: Enables patients to book, manage, or cancel appointments around the clock, including outside office hours.
- Digital anamnesis: Software-supported collection of patient data and forms (e.g., consent forms, information sheets) that can be completed digitally in advance or on site (e.g., via smartphone or tablet).
- Video consultation: Enables real-time telemedical consultations between physicians and patients via secure video services, without an in-person visit.
- Electronic patient record (ePA): A digital record for medical findings and information that patients can create with their health insurance provider. Patients control which data are stored and shared.

Which digital health solution would you like to consider in this survey?<br>Requirement: You have already used this solution. If multiple options apply, please choose the one you have used most often.	Online appointment booking
	Digital anamnesis
	Video consultation
	Electronic patient record
	None of the above

Use of Selected Digital Health Solution

Selected Digital Health Solution: [*Name of selected Digital Health Solution*]

Next, we would like to ask you about your current use of the selected solution.

Since when have you been using the selected solution?	I do not use it
	Less than 3 months
	3 to less than 6 months
	6 to less than 12 months
	1 year or more

How frequently have you used the selected solution within the last 12 months?	I do not use it
	For less than 20% of my medical appointments
	For 20% to less than 40% of my medical appointments
	For 40% to less than 60% of my medical appointments
	For 60% to less than 80% of my medical appointments
	For more than 80% of my medical appointments

Questions on the Effects of the Selected Solution on Service Quality_________

In this study, we use established service quality questionnaires. We focus on interactions between the outpatient practice and the patient before, during, and after treatment.

The following dimensions of service quality are assessed:

- General patient satisfaction
- Accessibility of the practice
- Improvement of health
- Appearance of the practice
- Empathy toward patients
- Information and communication management
- Organizational processes and efficiency
- Sense of safety and trust

You will now see a series of statements regarding service quality in outpatient practices. To what extent do you agree with the following statements, with a focus on the digital health solution you selected?

Selected Digital Health Solution: [*Name of selected Digital Health Solution*]

Note: The questionnaire is being applied for the first time in parallel to four different digital health solutions. Therefore, some statements may appear less applicable to the selected solution.

General patient satisfaction

The digital health solution contributes to the fact that…

	Strongly disagree	Largely disagree	Somewhat disagree	Somewhat agree	Largely agree	Strongly agree	I don't know
…I perceive the treatment overall as ideal. (GS1)	☐	☐	☐	☐	☐	☐	☐
…I am dissatisfied with some aspects of my treatment. (GS2)	☐	☐	☐	☐	☐	☐	☐

Accessibility of the practice

The digital health solution contributes to the fact that…

	Strongly disagree	Largely disagree	Somewhat disagree	Somewhat agree	Largely agree	Strongly agree	I don't know
…the practice is easy to find. (AC1)	☐	☐	☐	☐	☐	☐	☐
…the practice's online information is easy to find. (AC2)	☐	☐	☐	☐	☐	☐	☐
…it is easy for me to navigate the online offerings of the practice. (AC3)	☐	☐	☐	☐	☐	☐	☐
…the waiting time for appointments is acceptable for me. (AC4)	☐	☐	☐	☐	☐	☐	☐
…I receive all necessary information in advance. (AC15	☐	☐	☐	☐	☐	☐	☐
…the practice team is easily accessible for me. (AC6)	☐	☐	☐	☐	☐	☐	☐
…I don't have to wait too long for a treatment if I have an urgent concern. (AC7)	☐	☐	☐	☐	☐	☐	☐

*Questions on the Effects of the Selected Solution on Service Quality*________

You will now see a series of statements regarding service quality in outpatient practices. To what extent do you agree with the following statements, with a focus on the digital health solution you selected?

Selected Digital Health Solution: [*Name of selected Digital Health Solution*]

Note: The questionnaire is being applied for the first time in parallel to four different digital health solutions. Therefore, some statements may appear less applicable to the selected solution.

Improvement of health

The digital health solution contributes to the fact that…

	Strongly disagree	Largely disagree	Somewhat disagree	Somewhat agree	Largely agree	Strongly agree	I don't know
…I perceive the medical treatment as appropriate. (IM1)	☐	☐	☐	☐	☐	☐	☐
…the practice team makes great efforts for the best possible care. (IM2)	☐	☐	☐	☐	☐	☐	☐
…my medical conditions improve through the treatment in the practice. (IM3)	☐	☐	☐	☐	☐	☐	☐
…my overall condition improves through the visit to the practice. (IM4)	☐	☐	☐	☐	☐	☐	☐
…the practice team strives to prevent related diseases through patient education. (IM5)	☐	☐	☐	☐	☐	☐	☐
…the practice team is committed to supporting me in preventing a recurrence of illness. (IM6)	☐	☐	☐	☐	☐	☐	☐
…my conditions improve through the treatment in this practice. (IM7)	☐	☐	☐	☐	☐	☐	☐

Appearance of the practice

The digital health solution contributes to the fact that…

	Strongly disagree	Largely disagree	Somewhat disagree	Somewhat agree	Largely agree	Strongly agree	I don't know
…the practice uses modern medical devices and digital health solutions. (TA1)	☐	☐	☐	☐	☐	☐	☐
…the members of the practice team possess advanced skills and knowledge. (TA2)	☐	☐	☐	☐	☐	☐	☐
…my experience is comfortable both in the practice and online. (TA3)	☐	☐	☐	☐	☐	☐	☐
…the practice team always makes a clean and tidy impression both in the practice and online. (TA4)	☐	☐	☐	☐	☐	☐	☐
…the overall impression that I get of the practice, both in the practice rooms and online, is clean and tidy. (TA5)	☐	☐	☐	☐	☐	☐	☐

2+2=	[Free text field]

Questions on the Effects of the Selected Solution on Service Quality________

You will now see a series of statements regarding service quality in outpatient practices. To what extent do you agree with the following statements, with a focus on the digital health solution you selected?

Selected Digital Health Solution: [*Name of selected Digital Health Solution*]

Note: The questionnaire is being applied for the first time in parallel to four different digital health solutions. Therefore, some statements may appear less applicable to the selected solution.

Empathy toward patients

The digital health solution contributes to the fact that…

	Strongly disagree	Largely disagree	Somewhat disagree	Somewhat agree	Largely agree	Strongly agree	I don’t know
…the practice team shows a polite attitude. (EM1)	☐	☐	☐	☐	☐	☐	☐
…the practice team takes the time to explain the details. (EM2)	☐	☐	☐	☐	☐	☐	☐
…the practice team takes the time to listen to me. (EM3)	☐	☐	☐	☐	☐	☐	☐
…the practice team understands and considers my situation. (EM4)	☐	☐	☐	☐	☐	☐	☐
…the interactions of the practice team with me are characterized by closeness and friendliness. (EM5)	☐	☐	☐	☐	☐	☐	☐
…the practice team understands what I want. (EM6)	☐	☐	☐	☐	☐	☐	☐
…the practice team empathizes with my problems. (EM7)	☐	☐	☐	☐	☐	☐	☐
…the practice team takes enough time for the conversation. (EM8)	☐	☐	☐	☐	☐	☐	☐
…I understand the members of the practice team. (EM9)	☐	☐	☐	☐	☐	☐	☐

Information and communication management

The digital health solution contributes to the fact that I receive information about…

	Strongly disagree	Largely disagree	Somewhat disagree	Somewhat agree	Largely agree	Strongly agree	I don’t know
…self-care. (IN1)	☐	☐	☐	☐	☐	☐	☐
…medication and their side effects. (IN2)	☐	☐	☐	☐	☐	☐	☐
…planned examinations. (IN3)	☐	☐	☐	☐	☐	☐	☐
…the results of examinations and tests. (IN4)	☐	☐	☐	☐	☐	☐	☐
…my condition and prognosis. (IN5)	☐	☐	☐	☐	☐	☐	☐

*Questions on the Effects of the Selected Solution on Service Quality*________

You will now see a series of statements regarding service quality in outpatient practices. To what extent do you agree with the following statements, with a focus on the digital health solution you selected?

Selected Digital Health Solution: [*Name of selected Digital Health Solution*]

Note: The questionnaire is being applied for the first time in parallel to four different digital health solutions. Therefore, some statements may appear less applicable to the selected solution.

Organizational processes and efficiency

The digital health solution contributes to the fact that…

	Strongly disagree	Largely disagree	Somewhat disagree	Somewhat agree	Largely agree	Strongly agree	I don't know
…the practice team strives to avoid unnecessary medications. (EF1)	☐	☐	☐	☐	☐	☐	☐
…the practice team strives to find the most appropriate treatment method. (EF2)	☐	☐	☐	☐	☐	☐	☐
…the practice team makes the treatment procedures comfortable for me. (EF3)	☐	☐	☐	☐	☐	☐	☐
…the practice team strives to avoid unnecessary treatments and tests. (EF4)	☐	☐	☐	☐	☐	☐	☐
…the practice team provides sufficient background information to me both in the practice and online. (EF5)	☐	☐	☐	☐	☐	☐	☐
…the practice team works well together. (EF6)	☐	☐	☐	☐	☐	☐	☐
…the work in the practice is well organized. (EF7)	☐	☐	☐	☐	☐	☐	☐
…the practice team enters the interaction with well prepared. (EF8)	☐	☐	☐	☐	☐	☐	☐

Sense of safety and trust

The digital health solution contributes to the fact that…

	Strongly disagree	Largely disagree	Somewhat disagree	Somewhat agree	Largely agree	Strongly agree	I don't know
…the practice team creates a comfortable and safe environment for my treatment. (SA1)	☐	☐	☐	☐	☐	☐	☐
…I trust the doctors not to make misdiagnoses. (SA2)	☐	☐	☐	☐	☐	☐	☐
…I trust the medical assistants not to make mistakes. (SA3)	☐	☐	☐	☐	☐	☐	☐
…I trust the medical proficiency of the practice team. (SA4)	☐	☐	☐	☐	☐	☐	☐
…I feel safe from infections in the practice. (SA5)	☐	☐	☐	☐	☐	☐	☐
…the practice team creates a pleasant and safe atmosphere in all interactions. (SA6)	☐	☐	☐	☐	☐	☐	☐
…the practice team takes enough time to check everything when examining and treating me. (SA7)	☐	☐	☐	☐	☐	☐	☐
…I feel that my personal data is stored and processed safely in the practice. (SA8)	☐	☐	☐	☐	☐	☐	☐

Sociodemographic Information

Finally, we would like to ask you a few questions about yourself.

How large is your place of residence?<br>I live in a village/city with…	…less than 5,000 inhabitants.
	…5,000 to <20,000 inhabitants.
	…20,000 to <100,000 inhabitants.
	…100,00 to <500,000 inhabitants.
	…500,000 inhabitants or more.

How are you insured for healthcare?	I have a statutory healthcare insurance.
	I have I have a statutory healthcare insurance and a supplementary private insurance.
	I have a private healthcare insurance.
	I don’t have a healthcare insurance.

Which gender do you identify with?	Male
	Female
	Diverse
	Other

How old are you?	Younger than 26 years old
	26 to 35 years old
	36 to 45 years old
	46 to 55 years old
	56 to 65 years old
	Older than 65 years old

How digitally affine do you consider yourself?	Not at all digitally savvy
	Rather not digitally savvy
	Neither not digitally savvy nor digitally savvy
	Rather digitally savvy
	Fully digitally savvy

Closing

If you would like to complete the survey for another digital health solution, click here.

You have reached the end of the survey. We sincerely thank you for your participation.

You may now close the survey.
